# Screening of Reference miRNA of Different Early- and Late-Flowering Tree Peony Varieties

**DOI:** 10.3390/plants12142629

**Published:** 2023-07-12

**Authors:** Jiajia Shen, Xiaohui Wang, Yuying Li, Lili Guo, Xiaogai Hou

**Affiliations:** 1 College of Agriculture, Henan University of Science and Technology, Luoyang 471023, China; 2 Luoyang Academy of Agricultural and Forestry Sciences, Luoyang 471002, China

**Keywords:** miRNA, qRT-PCR, reference gene, different varieties, tree peony

## Abstract

miRNA plays an important role in plant growth and development and in response to various stresses. Quantitative real-time PCR (qRT-PCR) technology is often used to detect the expression level of miRNAs and genes by comparing with reference genes. In order to screen out the optimal reference miRNAs in different tree peony varieties, the petals of 42 different early- and late-flowering tree peony varieties were used as experimental materials, and geNorm, NormFinder, Bestkeeper, and RefFinder software were used to evaluate the stability of 16 candidate reference miRNAs. The results showed that the average Ct values of all candidate reference miRNAs were between 15.34 ± 0.29 and 32.64 ± 0.38. The optimal number of reference miRNAs was four, which were *PsPC-5p-19095*, *PsPC-3p-51259*, *PsmiR159a*, and *PsPC-3p-6660* in geNorm. The stability of *PsPC-3p-6660* was the highest in the analysis results of NormFinder software. Among the analysis results of Bestkeeper software, *PsMIR319-p5* has the highest stability. Among the results of comprehensive evaluation and analysis of several software using RefFinder, the candidate reference miRNA with the highest stability was *PsPC-3p-6660*. When *PsPC-3p-6660* was used as the reference miRNA, the expression of *PomiR171* and *PomiR414* in response to different flowering times of tree peony was relatively stable in 42 tree peony varieties, indicating that *PsPC-3p-6660* was stable and reliable. The results of this study provide a reference miRNA for studying the expression changes of miRNA in different tree peony varieties and further exploring the regulatory mechanism of miRNA in different peony varieties.

## 1. Introduction

Tree peony (*Paeonia suffruticosa* Andrews.) is a perennial woody plant, which belongs to Paeoniaceae, *Paeonia*, section Moutan. As a kind of multi-purpose plant with economic value, tree peony has high ornamental value because of its rich varieties, gorgeous colors, and diverse flowers [1,2]. In addition, it also has high nutritional and medicinal values because its root can be used as medicine and the seeds can be used to extract oil, which is beneficial to human health [3,4,5,6]. The natural flowering period of this tree is around April, which is a one-flower-a-year plant, and a few varieties have the phenomenon of second flowering [7,8]. Among them, middle-flowering tree peony varieties dominate, and early-flowering and late-flowering varieties are few. The characteristics of a relatively short and concentrated flowering period seriously affect the ornamental value of tree peony and limit the development of tree peony industry. Therefore, understanding the internal molecular mechanism of tree peony flowering may be helpful to discover new genes related to flowering traits, which can be used to regulate the flowering time of tree peony, prolong the group flowering period, and increase economic benefits.

At present, the research on tree peony mainly focuses on the construction of tissue culture rapid propagation system [9], cultivation and domestication [10], genetic diversity [11], seed oil analysis [12], and gene cloning [13]. In recent years, with the rapid development of biotechnology, transcriptome analysis [14], miRNA identification [15], and other related molecular biology studies have been gradually carried out, and gene expression correlation studies have gradually become the focus of research.

Gene expression analysis is an important tool to elucidate complex regulatory processes, such as genetics, signal transduction, and metabolic pathways in the plant life cycle [16]. Real-time quantification PCR can be used to verify gene expression levels and the reliability of sequencing results [17,18]. Compared with conventional real-time PCR (RT-PCR), quantitative real-time PCR (qRT-PCR) has the characteristics of high sensitivity, strong specificity, and low cost and has been widely used in gene expression level analysis [19,20]. However, the stability and accuracy of qRT-PCR results are affected by many factors, among which the reference gene plays a role in the correction and standardization of the results and is one of the important factors affecting the reliability of qRT-PCR results. Transcription levels of reference genes may change in different species, tissues, treatments, and developmental stages [21]. Therefore, it is necessary to select the most suitable reference gene according to the test requirements. At present, some software and algorithms for evaluating the stability of reference genes have been developed, such as geNorm, NormFinder, and Bestkeeper, and these algorithms have been successfully used to screen out the reference genes related to biotic and abiotic stress, growth, and development in plants, such as *Zea mays* [22], *Hylocereus* sp. [23], *Allium sativum* [24], and Itoh peony [25].

With the application of high-throughput sequencing and other technologies, a large number of microRNAs (miRNAs) have been found in many higher plants. miRNA is a class of small single-stranded non-coding RNA molecules with a length of about 20–24 nucleotides that regulate gene expression at the post-transcriptional level through sequence complementation [26]. The first plant miRNA was first discovered in *Arabidopsis* in 2002 [27]. Studies have shown that miRNAs have been reported to exist widely in a variety of plants, and miRNAs are involved in regulating plant growth and development, morphogenesis, and environmental stress response, such as drought, salt, and temperature [28,29]. It has been found that miRNAs play an important role in the flowering mechanism by regulating the expression of flowering genes [30]. For example, miR172 induced rice flowering by inhibiting the expression of *OsIDS1* and *SNB* of the target gene AP2 family [31]. miR159 could delay the flowering time by reducing the expression of the target gene MYB transcription factor in *Arabidopsis* [32]. Overexpression of miR171 could inhibit the expression of the target gene *Scarecrow*-like (*SCL*), which in turn leads to a late-flowering phenotype in Hordeum vulgare transgenic plants, accompanied by a smaller and translucent anther phenotype [33]. Therefore, it is also of great significance to explore miRNA related to tree peony flowering so as to realize the regulation of tree peony flowering. However, there was only one report on reference miRNA in tree peony for different bud development processes, flower development processes, and different tissues [34], but the screening of reference miRNA among different early- and late-flowering tree peony varieties has not been reported.

In this study, the petals of 42 different early- and late-flowering tree peony varieties were used as experimental materials. Primers of *U6* (snRNA) and 15 miRNAs from small RNA sequencing were designed, resulting in a total of 16 candidate genes selected as candidate reference genes for miRNA expression normalization. qRT-PCR was used to detect and analyze the expression stability of 16 candidate reference miRNAs in 42 different tree peony varieties. The stability of 16 candidate genes was evaluated by geNorm, NormFinder, Bestkeeper, and RefFinder, and the optimal reference miRNA was verified. The results of this study will provide reference miRNAs for the expression of other target miRNAs in different tree peony varieties.

## 2. Results

### 2.1. miRNAs Quality Analysis

The miRNAs of 42 different early- and late-flowering tree peony varieties were extracted from the petals. The integrity of miRNAs was detected by 3% agarose gel electrophoresis, and the concentration and purity of miRNAs were detected by NanoDrop 1000 spectrophotometer (Implen, Munchen, Germany). The miRNA of each sample had a single bright and clear band at about 100 bp, indicating that the integrity of miRNAs was good. The NanoDrop 1000 spectrophotometer detection results showed that the A_260/280 nm_ of each miRNA sample was 1.8–2.1, indicating that the purity of miRNAs was good, which met the requirements of subsequent tests.

### 2.2. Primers Specificity Analysis of 16 Candidate miRNAs from Different Tree Peony Varieties

**Figure 1:**
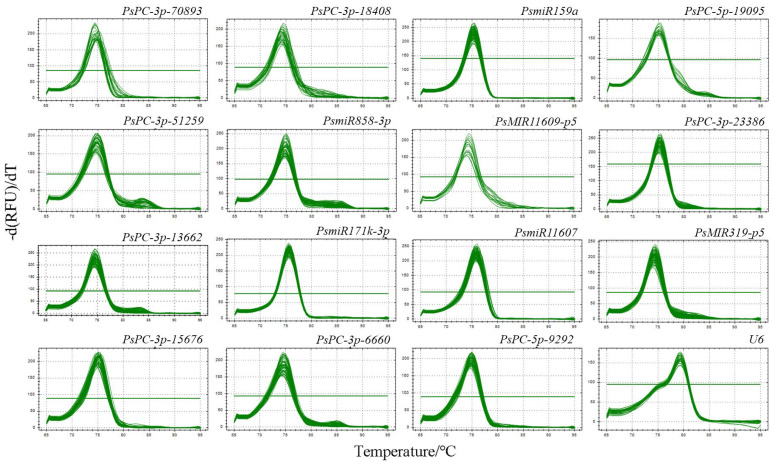
Primer melting curves analysis of 16 candidate reference miRNAs in different tree peony varieties. A single peak indicates that the primer specificity is good.

The primer melting curves of 16 candidate reference miRNAs were analyzed by qRT-PCR, and the results showed that the melting curves of all primers showed a single peak, indicating that the primers of the 16 candidate reference miRNAs had good specificity and could be used for the evaluation of the reference miRNAs (Figure 1).

### 2.3. Ct Value Analysis of 16 Candidate miRNAs of Different Tree Peony Varieties

**Figure 2:**
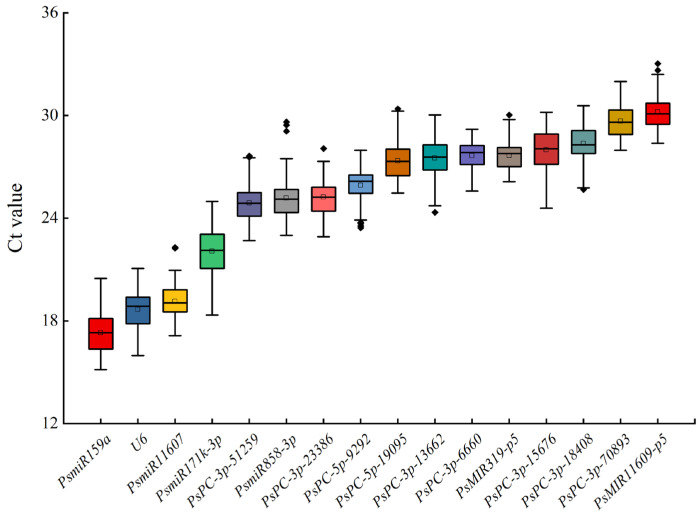
Expression levels of 16 candidate reference miRNAs in different tree peony varieties. The part of the box is the concentrated range of the Ct value, the ‘□’ in the box is the average, the ‘◈’ in the box is the outliers, the horizontal line in the box is the median, and the upper and lower side lines of the box are the upper four digits and the lower four digits, respectively.

The expression levels of miRNAs in 16 candidate reference miRNAs were analyzed by qRT-PCR. The results showed that the average Ct values of 16 candidate reference miRNAs in the petals of 42 tree peony varieties ranged from 15.34 ± 0.29 to 32.64 ± 0.38. The average Ct values in descending order were *PsmiR159a*, *U6*, *PsmiR11607*, *PsmiR171k-3p*, *PsPC-3p-51259*, *PsmiR858-3p*, *PsPC-3p-23386*, *PsPC-5p-9292*, *PsPC-5p-19095*, *PsPC-3p-13662*, *PsPC-3p-6660*, *PsMIR319-p5*, *PsPC-3p-15676*, *PsPC-3p-18408*, *PsPC-3p-70893*, and *PsMIR11609-p5*. *PsmiR159a* had the lowest average Ct value, indicating that it had the highest transcription level. *PsmiR11609-p5* had the highest average Ct value, indicating that it had the lowest transcription level. *PsmiR171k-3p* had the largest difference in the Ct value of a single gene among different samples, while *PsPC-3p-6660* had the smallest difference in the Ct value (Figure 2).

### 2.4. The Expression Stability of 16 Candidate Reference miRNAs Analyzed by geNorm

**Figure 3:**
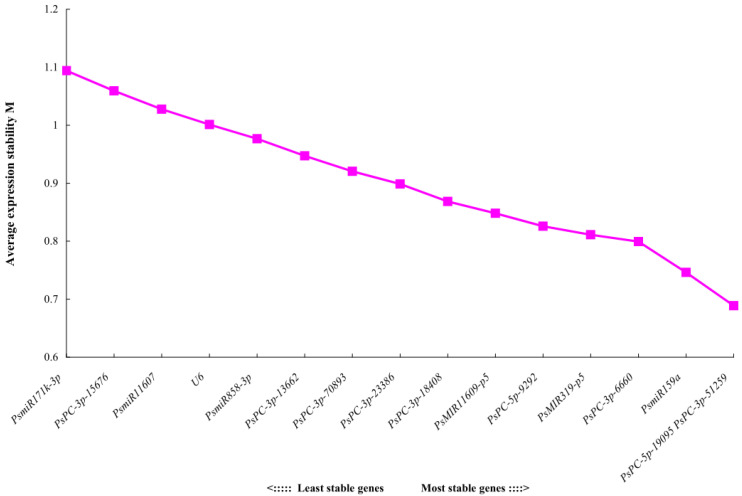
Expression stability values M of 16 candidate reference miRNAs analyzed by geNorm.

**Figure 4:**
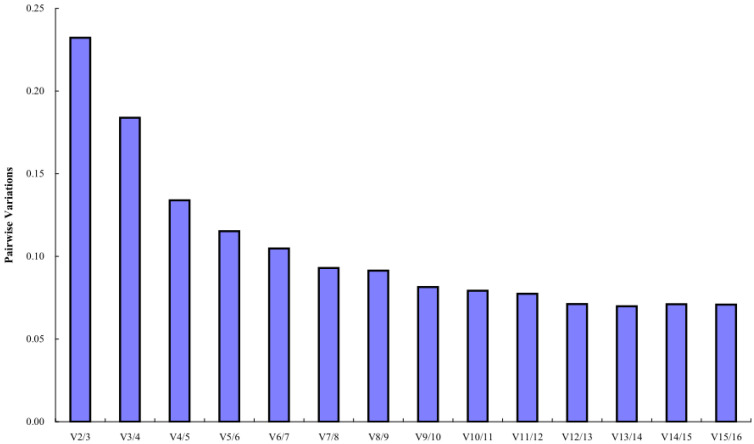
Pairwise variations of 16 candidate reference miRNAs.

The geNorm software mainly analyzes the expression stability of candidate reference genes through the expression stability M. The larger the M value, the more unstable the gene, and the smaller the M value, the more stable the gene. The analysis results showed that the stability order from high to low of 16 candidate reference miRNAs was *PsPC-5p-19095* = *PsPC-3p-51259* > *PsmiR159a* > *PsPC-3p-6660* > *PsMIR319-p5* > *PsPC-5p-9292* > *PsMIR11609-p5* > *PsPC-3p-18408* > *PsPC-3p-23386* > *PsPC-3p-70893* > *PsPC-3p-13662* > *PsmiR858-3p* > *U6* > *PsmiR11607* > *PsPC-3p-15676* > *PsmiR171k-3p*, indicating that *PsPC-5p-19095* and *PsPC-3p-51259* had the best stability, and the most unstable expression was *PsmiR171k-3p* (M = 1.09) (Figure 3). Pairwise variations can be used to determine the optimal number of reference genes. The results of geNorm histogram analysis showed that V_4/5_ = 0.13 < 0.15, indicating that the optimal number of references for miRNA quantitative expression was 4, which were *PsPC-5p-19095*, *PsPC-3p-51259*, *PsmiR159a*, and *PsPC-3p-6660* (Figure 4).

### 2.5. The Expression Stability of 16 Candidate Reference miRNAs Analyzed by NormFinder

NormFinder can rank the stability of candidate reference genes according to the stability value S of gene expression. NormFinder can not only compare differences between candidate genes but also calculate variations between sample groups. The smaller the S value, the more stable the gene, otherwise the worse the stability of the reference gene. According to the evaluation results of NormFinder software, the most stable expression of the 16 candidate reference miRNAs was *PsPC-3p-6660* (S = 0.37), followed by *PsPC-5p-19095* (S = 0.39), and the most unstable was *PsmiR171k-3p* (S = 0.79) (Table 1). The expression stability of 16 candidate reference genes in different early- and late-flowering tree peony varieties was ranked from high to low as follows: *PsPC-3p-6660*, *PsPC-5p-19095*, *PsMIR319-p5*, *PsPC-3p-51259*, *PsPC-5p-9292*, *PsMIR11609-p5*, *PsPC-3p-18408*, *PsmiR159a*, *PsPC-3p-23386*, *PsPC-3p-70893*, *PsPC-3p-13662*, *U6*, *PsmiR858-3p*, *PsmiR11607*, *PsPC-3p-15676*, and *PsmiR171k-3p*. Because NormFinder software can only screen out one best reference gene, *PsPC-3p-6660* was selected as the best reference base in the analysis using NormFinder software. The results were similar to geNorm software but with slight differences.

### 2.6. The Expression Stability of 16 Candidate Reference miRNAs Analyzed by Bestkeeper

The Bestkeeper software mainly evaluates the stability of the reference genes by calculating the standard deviation (SD) and the covariance (CV). The smaller the SD value, the better the gene stability. The analysis results showed that the SD values of most candidate miRNAs were less than 1, among which *PsMIR319-p5* had the best stability, followed by *PsPC-33-6660*. Only one candidate miRNA had an SD value greater than 1, namely, *PsmiR171k-3p*, indicating that it had the lowest stability (Table 2).

### 2.7. The Expression Stability of 16 Candidate Reference miRNAs Analyzed by RefFinder

Since the analysis results of different software have certain differences, the RefFinder software can be used for the final comprehensive analysis. RefFinder analysis is a comprehensive evaluation and ranking of the stability of reference genes obtained by geNorm, NormFinder, and Bestkeeper software. The analysis results showed that there were great similarities between the results obtained by RefFinder and the other three software, but there were also some differences. Among the petals of 42 different early- and late-flowering tree peony varieties, *PsPC-3p-6660* was the most stable and consistent with the results of NormFinder, followed by *PsPC-5p-19095*. *PsmiR171k-3p* was the most unstable miRNA, and the stability order from high to low is as follows: *PsPC-3p-6660*, *PsPC-5p-19095*, *PsMIR319-p5*, *PsPC-3p-51259*, *PsPC-5p-9292*, *PsMIR11609-p5*, *PsmiR159a*, *PsPC-3p-23386*, *PsPC-3p-18408*, *PsPC-3p-70893*, *PsmiR11607*, *PsPC-3p-13662*, *PsmiR858-3p*, *U6*, *PsPC-3p-15676*, and *PsmiR171k-3p*. There was consistency with the most unstable miRNA obtained by the other three software, indicating that the results were reliable (Table 3).

### 2.8. Validation of Reference miRNAs

**Figure 5:**
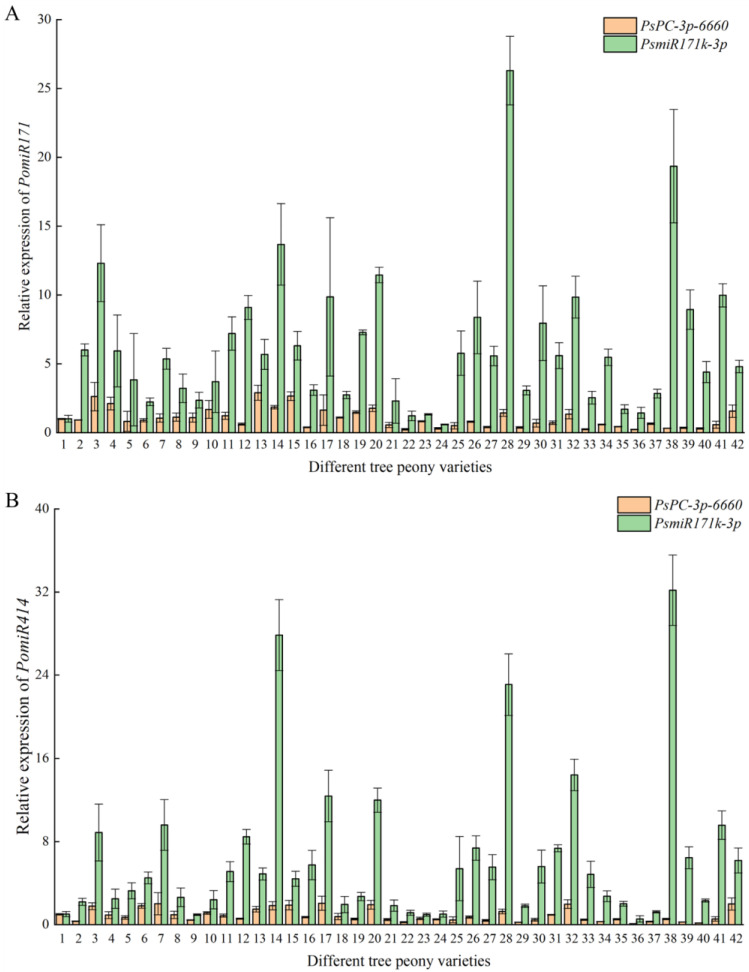
Expression analysis of *PomiR171* (**A**) and *PomiR414* (**B**) in different tree peony varieties using reference miRNAs (*PsPC-3p-6660* and *PsmiR171k-3p*). The numbers 1–42 represent 42 tree peony varieties.

In order to verify the reliability of the selected reference miRNAs, we selected the most stable reference, *PsPC-3p-6660*, and the most unstable reference, *PsmiR171k-3p*, as reference miRNAs to analyze the expression patterns of *PomiR171* and *PomiR414* in response to different flowering times in different tree peony varieties. The results showed that when *PsPC-3p-6660* was used as the reference miRNA, the relative expression levels of *PomiR171* and *PomiR414* in different tree peony varieties were relatively stable, and the average Ct values of 42 petal samples were 0.24 ± 0.03~2.91 ± 0.55 and 0.08 ± 0.03~2.06 ± 0.68, respectively. When *PsmiR171k-3p* was used as the reference miRNA, the relative expression levels of *PomiR171* and *PomiR414* in different tree peony varieties were quite different. The average Ct values of 42 petal samples were 0.60 ± 0.03~26.30 ± 2.49 and 0.56 ± 0.31~32.18 ± 3.40, respectively (Figure 5). Since *PomiR171* and *PomiR414* are differentially expressed miRNAs in response to different flowering stages of tree peony, when *PsPC-3p-6660* with high stability was used as the reference miRNA, the results of relatively stable expression of *PomiR171* and *PomiR414* among the samples were more reliable. When *PsmiR171k-3p* with the lowest stability was used as the reference miRNA, the results of relatively stable expression of *PomiR171* and *PomiR414* among the samples were not reliable. Therefore, *PsPC-3p-6660* is the most suitable reference miRNA of different early- and late-flowering tree peony varieties.

## 3. Discussion

Because the differences between species, materials, and tissues will affect the stability and reliability of quantitative results, it is necessary to select appropriate reference genes for data standardization during qRT-PCR. In this study, 42 early- and late-flowering tree peony varieties were used as materials, and the best reference miRNA was successfully screened out, which provided suitable reference miRNA for miRNA research of different tree peony varieties.

In general, the same reference does not have stability all the time. Under drought stress, the most suitable reference miRNAs in roots and leaves of *Glycine max* were miR156a and miR167a [35]. In *Allium sativum*, the most stable reference miRNA for different explants was *AsmiR168a-5p* and for different genotypes, the most stable reference miRNA was *AsmiR159a-1* [36]. In *Juglans regia*, the most suitable reference miRNAs for flower buds at different differentiation stages were *jre-miR394a*, *jre-miR159a*, and *jre-miR159c*, and the most suitable reference miRNAs for leaf buds at different differentiation stages were 5.8S rRNA and *jre-miRn3* [37]. The most stable reference miRNA combinations during seed development in *Brassica napus* were *miR167-1_2*, *miR11-1*, and *miR159-1* [38].

In this study, the stability analysis of 16 candidate reference miRNAs in 42 different tree peony varieties showed that *PsPC-5p-19095* and *PsPC-3p-51259* had the highest stability in the geNorm software. *PsPC-3p-6660* had the highest stability in the NormFinder software. *PsMIR319-p5* has the highest stability in the Bestkeeper software. In the results of comprehensive evaluation and analysis using RefFinder, the candidate reference miRNA with the highest stability was *PsPC-3p-6660*, which was consistent with the results of NormFinder and slightly different from the analysis results of geNorm and Bestkeeper software. The difference between the analysis results of each software has also appeared in the previous research results [39]. The reason for the difference in the stability of each candidate reference miRNA may be due to the large number of candidate reference miRNAs selected. Secondly, the difference in mathematical algorithms between different software will also affect the stability ranking of the test results to a certain extent. The results of several software showed that the stability of *PsmiR171k-3p* was the lowest, indicating that the candidate miRNA was not suitable as the reference gene in different tree peony varieties.

At present, *U6* is one of the most common reference genes, which is used as the reference gene in miRNA quantitative expression analysis of various plants, such as *Vitis vinifera* [40], *Brassica oleracea* [41], and *Jatropha curcas* [42]. Studies have shown that *U6* is not stable in all cases [43]. *U6* is a suitable reference miRNA for different tissues and stem tissues under drought stress in *Hylocereus polyrhizus* [44]. However, in the evaluation of the expression stability of walnut flower bud and leaf bud differentiation, tissue parts, and varieties, *U6* had the worst stability and was not suitable as the reference miRNA. This phenomenon also exists in plants, such as wheat and longan [45]. In this study, *U6* was used as a candidate reference miRNA, and its stability was low in the four software, which was not suitable as the reference miRNA for different tree peony varieties.

## 4. Materials and Methods

### 4.1. Plant Materials

A total of 42, i.e., 21 early-flowering and 21 late-flowering, tree peony varieties were selected from the experimental farm of Henan University of Science and Technology (112°24′52.05″ E, 34°35′45.91″ N) as experimental material for the evaluation of the expression stability of candidate reference genes from April to May 2021. The samples were frozen with liquid nitrogen and then stored in a −80 °C refrigerator for later use (Table 4).

### 4.2. Primers Design of Candidate Reference miRNAs

In this study, *U6* and 15 miRNAs with relatively stable expression levels were selected from the small RNA sequencing data of *Paeonia ostii* ‘Fengdan’, Mutant plants of *Paeonia ostii* ‘Fengdan’, and *Paeonia suffruticosa* ‘Lianhe’ (CNGBdb, accession number CNP0002984) at the blooming stage (BS), initial flowering stage (IF), full blooming stage (FB), and decay stage (DE) in our laboratory, including 6 known miRNAs (*PsmiR159a*, *PsmiR858-3p*, *PsMIR11609-p5*, *PsmiR171k-3p*, *PsmiR11607*, and *PsMIR319-p5*) and 9 novel miRNAs (*PsPC-3p-70893*, *PsPC-3p-18408*, *PsPC-5p-19095*, *PsPC-3p-51259*, *PsPC-3p-23386*, *PsPC-3p-13662*, *PsPC-3p-15676*, *PsPC-3p-6660*, and *PsPC-5p-9292*).

qRT-PCR primers were designed using poly (A) by Primer Premier 5.0 software. Primers should avoid special structures such as dimers and hairpin structures to prevent adverse effects on test results. Then, they were sent to Sangon Biotech (Shanghai) Co., Ltd. (Shanghai, China) for synthesis (Table 5).

### 4.3. Isolation of miRNA and Synthesis of cDNA

miRNAs were isolated from the petals of 42 tree peony varieties using the miRcute Plant miRNA Isolation Kit (Tiangen, Beijing, China). Then, miRcute Plus miRNA First-Strand cDNA Synthesis Kit (Tiangen, China) was used to synthesize the first strand. Reaction system (20 μL): Total miRNA 2 μL, 2× miRNA RT Reaction Buffer 10 μL, miRNA RT Enzyme Mix 2 μL, and RNase-free ddH_2_O 6 μL. The reaction procedure was as follows: 42 °C for 60 min and 95 °C for 3 min.

### 4.4. Primers Specificity Analysis

Using cDNA as a template, qRT-PCR primers were used for PCR amplification using a 2× PCR Taq Master Mix (Blue Dye) (Nobelab Biotech, Beijing, China). Reaction system (25 μL): 2× PCR Taq Master Mix 12.5 μL, Primer F (10 μM) 1 μL, Primer R (10 μM) 1 μL, cDNA 2 μL, ddH_2_O 8.5 μL. The reaction procedure was as follows: 95 °C for 2 min; 95 °C 30 s, 60 °C 30 s, 72 °C 10 s, 35 cycles; 72 °C for 2 min.

cDNAs were diluted in a gradient of 5^1^, 5^2^, 5^3^, 5^4^, and 5^5^. An SYBR^®^ Green Premix Pro *Taq* HS qPCR Kit (Accurate Biology, Changsha, China) was used for qRT-PCR. Reaction system (20 μL): 2× SYBR^®^ Green Pro *Taq* HS Premix 10 μL, Primer F (10 μM) 0.4 μL, Universal Primer R (10 μM) 0.4 μL, cDNA 2 μL, ddH_2_O 7.2 μL. The reaction procedure was as follows: 95 °C 30 s; 95 °C 5 s, 60 °C 30 s, 40 cycles. Three technique replicates were set for each sample.

### 4.5. Expression Stability Analysis of 16 Candidate Reference miRNAs

The stability of 16 candidate reference miRNAs was evaluated and ranked using geNorm, NormFinder, and Bestkeeper software, respectively. And then the online tool RefFinder (http://blooge.cn/RefFinder/, accessed on 13 February 2023) [46,47] was used to comprehensively evaluate and analyze the results obtained by the above three software. Finally, the most suitable reference miRNA among different tree peony varieties was screened.

### 4.6. Validation of Candidate Reference miRNAs

The most stable and the unstable miRNAs were selected as the reference miRNAs, and the expression patterns of *PomiR171* and *PomiR414* (CNGBdb, accession number CNP0002984) in response to different flowering times of tree peony in different tree peony varieties were analyzed. Three technique replicates were set for each sample, and the qRT-PCR primers were given in Table 6. The expression levels of the miRNA were calculated using the formula of 2^−ΔΔCt^, and statistical analysis and mapping were performed using SPSS 22.1 (one-way ANOVA, Duncan’s test, *p* < 0.05) and Origin 2018.

## 5. Conclusions

Using petals of 42 different early- and late-flowering tree peony varieties as experimental materials, the expression stability of 16 candidate reference genes was evaluated and analyzed by geNorm, NormFinder, Bestkeeper, and RefFinder software using qRT-PCR. The results showed that the average Ct values of all candidate reference miRNAs were between 15.34 ± 0.29 and 32.64 ± 0.38. In the geNorm software, *PsPC-5p-19095* and *PsPC-3p-51259* had the highest stability, and the optimal number of reference miRNAs was four, which were *PsPC-5p-19095*, *PsPC-3p-51259*, *PsmiR159a*, and *PsPC-3p-6660*. The stability of *PsPC-3p-6660* was the highest in the analysis results of the NormFinder software. Among the analysis results of the Bestkeeper software, *PsMIR319-p5* had the highest stability. Among the results of comprehensive evaluation and analysis of several software using RefFinder, the candidate reference miRNA with the highest stability was *PsPC-3p-6660*. Therefore, *PsPC-3p-6660* can be used as the reference miRNA for miRNA studies of different tree peony varieties. This provides a reference miRNA for the study of other miRNAs in different tree peony varieties.

## Figures and Tables

**Table 1:** NormFinder analysis of 16 candidate reference miRNAs ranking.

miRNA	Stability Value	Standard Error	Rank
*PsPC-3p-6660*	0.37	0.03	1
*PsPC-5p-19095*	0.39	0.03	2
*PsMIR319-p5*	0.40	0.03	3
*PsPC-3p-51259*	0.42	0.03	4
*PsPC-5p-9292*	0.43	0.03	5
*PsMIR11609-p5*	0.44	0.03	6
*PsPC-3p-18408*	0.46	0.03	7
*PsmiR159a*	0.48	0.03	8
*PsPC-3p-23386*	0.54	0.04	9
*PsPC-3p-70893*	0.54	0.04	10
*PsPC-3p-13662*	0.60	0.04	11
*U6*	0.62	0.04	12
*PsmiR858-3p*	0.64	0.04	13
*PsmiR11607*	0.66	0.04	14
*PsPC-3p-15676*	0.72	0.05	15
*PsmiR171k-3p*	0.79	0.05	16

The smaller the Stability Value and Standard Error, the more stable the gene is.

**Table 2:** Bestkeeper analysis of 16 candidate reference miRNAs ranking.

miRNAs	SD	CV	Rank
*PsMIR319-p5*	0.59	2.15	1
*PsPC-3p-6660*	0.67	2.43	2
*PsmiR11607*	0.75	3.91	3
*PsPC-5p-19095*	0.76	2.76	4
*PsMIR11609-p5*	0.77	2.55	5
*PsPC-3p-23386*	0.77	3.04	6
*PsPC-3p-70893*	0.78	2.64	7
*PsPC-5p-9292*	0.80	3.09	8
*PsPC-3p-51259*	0.80	3.21	9
*PsmiR858-3p*	0.82	3.26	10
*PsPC-3p-18408*	0.84	2.97	11
*PsPC-3p-13662*	0.84	3.06	12
*PsmiR159a*	0.89	5.14	13
*U6*	0.95	5.11	14
*PsPC-3p-15676*	0.98	3.50	15
*PsmiR171k-3p*	1.10	4.99	16

The smaller the SD and CV values, the more stable the gene is.

**Table 3:** RefFinder analysis and ranking of 16 candidate reference miRNAs.

Rank	miRNAs	Geomean of Ranking Values
1	*PsPC-3p-6660*	1.68
2	*PsPC-5p-19095*	2.00
3	*PsMIR319-p5*	2.59
4	*PsPC-3p-51259*	3.36
5	*PsPC-5p-9292*	6.06
6	*PsMIR11609-p5*	6.24
7	*PsmiR159a*	7.07
8	*PsPC-3p-23386*	7.98
9	*PsPC-3p-18408*	8.10
10	*PsPC-3p-70893*	8.91
11	*PsmiR11607*	9.53
12	*PsPC-3p-13662*	11.24
13	*PsmiR858-3p*	11.93
14	*U6*	12.72
15	*PsPC-3p-15676*	15.00
16	*PsmiR171k-3p*	16.00

The smaller the Geomean of Ranking Values, the more stable the gene is.

**Table 4:** 42 tree peony varieties.

		Varieties (Number)	
Early-flowering tree peony varieties	*Paeonia suffruticosa* ‘Taoyanhong’ (1)	‘Huolianjindan’ (2)	‘Pinghuqiuyue’ (3)
	‘Jiaohong’ (4)	‘Dapengzhanchi’ (5)	‘Cangjiao’ (6)
	‘Lanju’ (7)	‘Haibo’ (8)	‘Lanhudie’ (9)
	‘Lanyueliang’ (10)	‘Zhaofen’ (11)	‘Yuncuicaidie’ (12)
	‘Jianshifen’ (13)	‘Xishifen’ (14)	‘Mantianxing’ (15)
	‘Jingyu’ (16)	‘Suxinbai’ (17)	‘Xueyuanhongxing’ (18)
	*Paeonia rockii* ‘Zibanbai’ (19)	‘Erqiao’ (20)	*Paeonia ostii* ‘Fengdan’ (21)
Late-flowering tree peony varieties	‘Feiyanhongzhuang’ (22)	‘Shouanhong’ (23)	‘Haitangzhengrun’ (24)
	‘Daduolan’ (25)	‘Doulv’ (26)	‘Shenheizi’ (27)
	‘Zihongzhengyan’ (28)	*Paeonia rockii* ‘Mingmou’ (29)	*Paeonia rockii* ‘Xianemao’ (30)
	*Paeonia rockii* ‘Baizhangbing’ (31)	*Paeonia rockii* ‘Baiyanwei’ (32)	*Paeonia rockii* ‘Yubanxiuqiu’ (33)
	‘Shuixinfenhe’ (34)	*Paeonia rockii* ‘Fenzouchou’ (35)	‘Xiuqiuhong’ (36)
	‘Zilouxiangcui’ (37)	‘Ziyan’ (38)	‘Jinge’ (39)
	‘Jinzhi’ (40)	‘Baiwangshizi’ (41)	‘Lianhe’ (42)

**Table 5:** Primers for qRT-PCR.

miRNAs	miRNA Sequence (5′-3′)	Primer F Sequence (5′-3′)
*PsPC-3p-70893*	TTCAACCCAACTTCGTCTCTT	CGCCTTCAACCCAACTTCGTC
*PsPC-3p-18408*	TTACGTTGCCTTTCTTCCTCTG	GGCGTTGCCTTTCTTCCTCTG
*PsmiR159a*	TTTGGATTTAAGGGAGCTCTA	GCGGGTTTGGATTGAAGGGAG
*PsPC-5p-19095*	AAAAGTCGGATCGCCAGCAACATC	CGTCGGATCGCCAGCAACAT
*PsPC-3p-51259*	AAATCTCGCCCAGACCCATG	CCAAATCTCGCCCAGACCCAT
*PsmiR858-3p*	CTCGTTGTCTGTTCGACCTTG	CCGTGTTGTCTGTCCGACCTTG
*PsMIR11609-p5*	TGAACCCTTTTTCCTACACT	CCGCCTGAACCCTTTTTCCTAC
*PsPC-3p-23386*	TGTGCTCTCCCTCTTCGTCAA	GCCTTGTGCTCTCCCTCTTCGT
*PsPC-3p-13662*	CGCCTCTTCCCTTGATTAAAC	GCCTTCGCCTCTTCCCTTGATT
*PsmiR171k-3p*	TTGAGCCGCGCCAATATCACT	CTCAGCCGCGCCAATATCAC
*PsmiR11607*	ACTCGGTTGTCTGACAGAC	GCTTGGCACTCGGTTGTCTGAC
*PsMIR319-p5*	CTGCCATCTCATGCATAAGGT	CGCCTGCCATCTCATCCATAAG
*PsPC-3p-15676*	AATCTCGTCCAGACCTATGGC	CGCCAATCTCGTCCAGACCTAT
*PsPC-3p-6660*	AACACGGGAAGTAGGCATTGCAGC	GGAACACGGGAAGTAGGCATTG
*PsPC-5p-9292*	CAACATCTTCGGCATCTAATCAGA	GCTGGCAACATCTTCGGCATCT
*U6*		ACAGAGAAGATTAGCATGGCC

**Table 6:** Primers for qRT-PCR.

miRNAs	miRNA Sequence (5′-3′)	Primer F Sequence (5′-3′)
*PomiR171*	TTGAGCCGCGTCAATATCTCT	GGTCAGCCGCGTCAATATCTCT
*PomiR414*	TCATCATCGTCATCATCTTCC	CCGCATCATCGTCATCATCTTCC

## Data Availability

The datasets generated during and/or analyzed during the current study are listed in the text and its additional files.
